# Cytotoxic, antioxidant, antibacterial activity of phytochemicals from *Phragmanthera austroarabica*

**DOI:** 10.6026/973206300200487

**Published:** 2024-05-31

**Authors:** Moodi S A. Alsubeie, Nasir A Ibrahim, Ahmed A Alghamdi, Nosiba S Basher, BS Al-ammari, Awadallah B Dafaallah, Vajid Nettoor Veettil

**Affiliations:** 1 Department of Biology, Faculty of Science, Imam Mohammed Ibn Saud Islamic University, Riyadh, Saudi Arabia; 2 National Center for Vegetation Cover Development and Combating Desertification (NCVC), Ministry of Environment, Water and Agriculture, Riyadh, Kingdom of Saudi Arabia; 3 Department of Crop Protection, Faculty of Agricultural Sciences, University of Gezira, Wad Medani, Sudan; 4 Iqraa Centre for Research and Development, IQRAA International Hospital and Research Centre, Kozhikode, Kerala, India; 5 MHES College of Science and Technology, Kozhikode, Kerala, India

**Keywords:** Cytotoxic effect, antioxidant, antibacterial activity, phytochemicals profile, *P. austroarabica*

## Abstract

The cytotoxic, antioxidant, anticancer, and antibacterial properties of ethanolic extracts from **Phragmanthera* austroarabica* is of interest. Plants of *P. austroarabica* were gathered from the southern Saudi Arabian region of Albaha. *P. austroarabica* extract was assessed using DPPH (2, 2-diphenyl-1-picrylhydrazyl). The German Collection of Microorganisms and Cell Cultures (DSMZ) cancer cell lines used in this investigation. The cytotoxic activity of *P. austroarabica* extract was explored against MCF-7 breast and A549 lung cancer cell lines, along with doxorubicin as a positive control. In both treated cells, *P. austroarabica* showed a remarkable activity *via* suppressing the cell's survival. In terms of IC50 (concentration equivalent to a survival rate of 50%), MCF-7 breast cancer cells were more sensitive to *P. austroarabica* extract.) DPPH colorimetric assay was employed to assess the antioxidant properties of *P. austroarabica* extract, the antioxidant activity was increased along with increment of extract concentrations. The leaves aqueous extract of *P. austroarabica* inhibited the growth of *S. aureus* by 6.3±0.12 mm and 24±0.43 mm and 15±0.56 mm respectively for seed, leaf and stem at concentrations 50 µl. However, the same concentrations inhibited the growth of *E. coli* by 25±0.75, 0.00 mm and 24±0.18 mm, following the same order. Different superscript letters indicate means that are significantly different at level (p<0.05). Minimal inhibitory concentrations (MIC) of *P. austroarabica* ethanolic extracts against the tested microorganisms were 1.5, 1.6 and 1.5, respectively for seed, leaf and stem against *Staph. Aureus* and were 1.2, 0.00 and 1.2, respectively for seed, leaf and stem against *E. coli*.

## Background:

Biodiversity from nature, particularly plants, is still the important source of medicinal products since the past century [1]. In the review by Newman and Cragg [2] it was stated that in the area of cancer drugs, from 155 small drugs molecules, 47% derived from or natural products itself. Therefore, exploration of new leads for drug discovery and development from plants is still important [3]. For the country like Saudi Arabia, that is rich in plant biodiversity, opportunity to find new leads for drug discovery need to be explored by investigating the bioactivities of plants that already used for traditional/alternative medicine. Mistletoes are semi-parasitic perennial flowering plants that also known as medicinal plants [4]. As a semi-parasitic plant, mistletoe is considered as an unwanted plant to economically important horticultural plant [5], however in the other side, mistletoe is known as one of medicinal plant used in traditional/alternative medicine several countries [6]. According to [7], mistletoes are a representative of several families in the order Santalales, particularly Loranthaceae (1044 species) and Viscaceae (570 species). Nine mistletoe species from five genera and two families were found in the Kingdom of Saudi Arabia. *Oncocalyx glabratus* (Engl.) MG Gilbert, *Oncocalyx schimperi* (Hochst. ex A. Rich.) MG Gilbert, *Phragmanthera austroarabica*, *Plicosepalus acaciae* (Zucc.) Wiens and Polhill, *Plicosepalus curviflorus* & *Tapinanthus globiferus* (A. Rich.) Tiegh. Are members of the Loranthaceae family. On the other hand, *Viscum cruciatum* Sieber ex Boiss, *Viscum triflorum* ssp. *nervosum D* and *Viscum schimperi* are members of the Viscaceae family [8] [9][10].

Mistletoes have been used in folk medicine for a very long time in many different forms. Mistletoe preparations are widely used in many cultures on almost all continents to treat or manage a variety of medical problems such as hypertension, diabetes mellitus, inflammatory conditions, irregular menstruation, menopause, epilepsy, arthritis, cancer and etc. [11]. Mistletoe preparations in the form of injectable extracts, infusions, tinctures, fluid extracts, or tea bags are widely used in many cultures on almost all continents [6]. Lectins and viscotoxins, two of the most researched and potent phytocomponents of mistletoe, are crucial in the treatment of cancer because of their cytotoxic and apoptotic effects. The immunomodulatory effects of both groups have been demonstrated [12]. The phenolic acids, phenylpropanoids, and flavonoids, which have antioxidant and anti-inflammatory properties and may reduce blood pressure, are another group of compounds found in mistletoe [13].

Triterpenic acids, in particular the cytotoxic and apoptotic oleanolic, ursolic, and betulinic acids, have also been discovered in mistletoe [14]. Other significant pharmacological substances found in mistletoe include phytosterols, alkaloids, oligopeptides, polysaccharides, and fatty acids [15]. Numerous variables relating to the environment and growing conditions, as well as the growth and development of the plant itself, have an impact on the accumulation of secondary metabolites in plants [16]. It has been demonstrated in the past that the type of tree on which mistletoe grows affects its metabolic profile [17]. Additionally, the biological activity of various plant parts can differ depending on the qualitative and quantitative composition of phytocomponents in those parts. It is necessary to clarify the differences between ethnomedical uses and modern pharmacology as well as between phytochemistry screening and structure elucidation. In-depth research should be done on the identification of bioactive compounds in crude extracts and fractions, the illustration of the underlying pharmacological mechanisms, as well as cytotoxicity, genotoxicity, and clinical trials of toxic tests [18]. Therefore, it is of interest to evaluate the cytotoxic, antioxidant, anticancer and antimicrobial activities of water extracts of Mistletoe [19] collected from Saudi Arabia.

## Materials and Methods:

## Plant material:

*Phragmanthera austroarabica* plants were collected from the Al-Baha region south-west of Saudi Arabia. The work carried out at Department of Biology Faculty of Science, Imam Mohammad Ibn Saud Islamic University (IMSIU), and Saudi Arabia. The plant materials (seeds, leaves and stems) were air-dried in shadow for 2 weeks then grounded into a powder and kept at room temperature (25°C) until used.

## Preparation of plant extract:

For ethanol extraction, 20 g of each of the air-dried powder was added to 100 ml of ethanol 70% and incubated for 24 hours on a shaker. Then it was filtered through 8 layers of muslin cloth and centrifuged at 5000 rpm for 10 minutes and the supernatant was collected. Then the concentrated to make the final volume one-fourth of the original volume (stock solution).

## Cytotoxic activity assessment of (MTT Assay):

The MTT assay was conducted following a previously described protocol (Nasr *et al.*, 2020). In summary, cells in their exponential growth phase were trypsinized, counted, and seeded into 96-well plates at a density of 50,000 cells per well in 100 µL of DMEM medium. After a 24-hour incubation period, the cells were exposed to varying concentrations of *P. austroarabica* extract (200, 100, 50, 25, and 0 µg/mL), with doxorubicin used as a positive control. Following 48-hour incubation, 10 µL of MTT solution (5 mg/mL in PBS) was added to each well and left for an additional 2-4 hours at 37°C. The resulting purple formazan product was dissolved using 100 µL of HCl isopropanol per well, and the plates were shaken for 10 minutes. The absorbance at 540 nm was measured using a plate reader (BioTek, USA). Dose-response curves were constructed to determine the IC_50_ (half-maximal concentration) values, which were calculated using OriginPro 8.5 software. The cell viability percentage was calculated as follows = [mean absorbance of the treated sample / mean absorbance of the control] x 100.

## Determination of antioxidant activity (DPPH assay):

Antioxidative activity of *P. austroarabica* extract was determined by DPPH (2, 2-diphenyl-1-picrylhydrazyl) assay as previously described [5]. Briefly, DPPH radical (0.2 mM) in methanol solution was prepared and 0.125 mL of this solution was added to 0.5 mL to plant extracts of different concentrations (1000, 500, 100, 50, 10 µg/ml). After 30 min of incubation period, the absorbance was taken at 517 nm. Scavenging action was calculated as follow: % of radical scavenging activity = (Abs control - Abs extract / Abs control) x 100

## Cell culture:

The breast (MCF-7; ACC115) and lung (A549; ACC107) cancer cell lines utilized in this study were obtained from the German Collection of Microorganisms and Cell Cultures (DSMZ), Braunschweig, Germany. The cells were cultured in DMEM medium supplemented with 10% FBS and 1% penicillin-streptomycin, which were procured from Gibco, Invitrogen, Thermo Fisher Scientific, and USA.

## Antibacterial activities of plant extracts:

Antibacterial activity of each ethanolic extracts of *P. austroarabica* parts were determined by agar diffusion method. Fresh isolated colony of *S. aureus* and *E. coli* were suspended in sterile saline to get turbidity of 0.5 McFarland standards. A quantity of 0.1 ml of this suspension was spread aseptically on sterile Muller Hinton agar medium (Hi media). The wells of 6 mm diameter were bored by sterile cork borer. A quantity of 0.2 ml of each extract (100 mg/ml in 10% Dimethyl sulfoxide; DMSO was added to the wells. It was allowed to diffuse by keeping in freeze for 20 minutes. Ten percent (10%) DMSO in one of the wells was used as negative control. After diffusion of each extract, the plates were incubated at 37°C for 24 hours. The inhibition zones were measured in mm. For each extract, three replicates were maintained.

## Determination of minimum inhibitory concentration (MIC):

Tube dilution method was done to determine minimum inhibitory concentration of each extract. A series of two-fold dilutions of each extract ranging from 10 mg/ml *S. aureus* and *E. coli* to 0.3 mg/ml were made in Muller Hinton broth. A quantity of 0.1 ml of suspension of *S. aureus* and *E. coli* matched to 0.5 McFarland standard was seeded into each dilution. Two controls were maintained for each test batch. These controls included tube containing extract and growth medium without inoculum and organism control i.e., tube containing the growth medium and inoculum. The tubes were incubated at 37°C for 24 hours and checked for turbidity. Minimum inhibitory concentration was determined as highest dilution of the extract that showed no visible growth.

## Gas chromatography-mass spectrometry (GC-MS) measurement:

GC-MS analysis was performed using a Perkin Elmer Clarus 600 GC coupled with a mass spectrometer (Turbomass). A 1 µL extract volume was injected into the Elite5MS column, which possessed dimensions of 30 m in length, a film thickness of 0.25 µm, and an internal diameter of 0.25 µm. The injection was executed under the prescribed temperature protocol. The gas chromatography-mass spectrometry (GC-MS) system begins by setting the initial oven temperature at 40°C and keeping it constant for 2 minutes. Following this, the temperature is increased to 200°C with a pace of 5°C per minute, and this heightened temperature is maintained for an additional duration of 2 minutes. Commencing at an initial temperature of 200°C, the temperature exhibits a linear progression with a rate of 5°C per minute, ultimately attaining a final value of 300°C. Following this, the temperature remains consistent at this particular level for two minutes. The temperature of the injector was held constant at 280 °C. The temperature of the interface was measured to be 240°C, although the source's temperature was recorded as 220°C. The system's vacuum pressure was maintained at a magnitude of 1.11 x 10^-5 torr, while the energy of the electrons was configured to be 70 electron volts (eV). In this experiment, helium was used as the mobile phase at a 1.0 mL/min flow rate. The mass spectra were obtained utilizing the electron ionization technique; with a scanning range from 40 to 600 m/z. Unidentified chemicals were discovered by comparing their spectra with those documented in the National Institute of Standard and Technology (2005) and WILEY (2006) libraries. The total time required to analyze a single sample was 58 minutes.

## Statistical analysis:

Data were recorded then plotted and statistically analyzed by using ANOVA one way to compare the mean± standard division of the tested sample.

## Results:

## Cytotoxicity of *P. austroarabica*:

**Figure 1:**
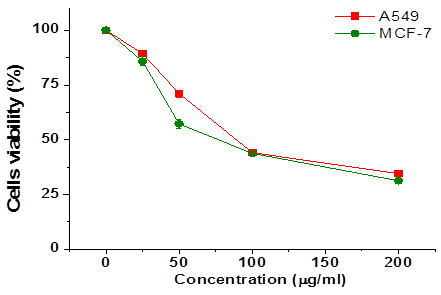
Cytotoxicity of *P. austroarabica* extract on human breast MCF-7 and lung A549 cancer cell lines assessed by using MTT assay. Values represent AVG ± SD of three independent experiments.

The cytotoxic activity of *P. austroarabica* extract was explored against MCF-7 breast and A549 lung cancer cell lines, along with doxorubicin as a positive control. In both treated cells, *P. austroarabica* showed a remarkable activity *via* suppressing the cell's survival (Figure 1). In terms of IC50 (concentration equivalent to a survival rate of 50%), MCF-7 breast cancer cells were more sensitive to *P. austroarabica* extract (Table 1).

## Antioxidant properties of *P. austroarabica*:

**Figure 2:**
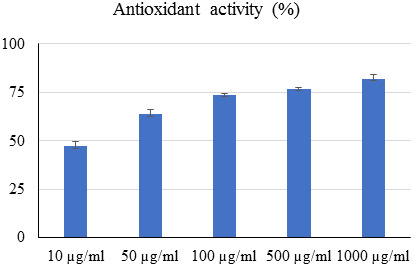
Antioxidant potential of *P. austroarabica* extract using DPPH assay. Values represent % radical scavenging (AVG ± SD) of three replicates.

DPPH colorimetric assay was employed to assess the antioxidant properties of *P. austroarabica* extract. As depicted in (Figure 2), the antioxidant activity was increased along with increment of extract concentrations.

## Antibacterial activity:

**Figure 3:**
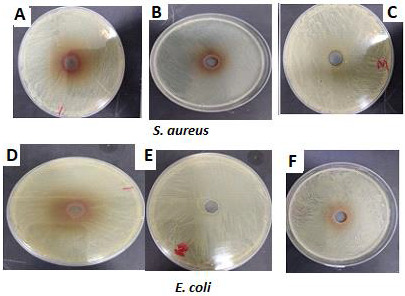
Antibacterial activity (mm) of *P. austroarabica* ethanolic extracts. A and D: Seed extract; B and E: leaf extract; C and F: stem extract.

**Figure 4:**
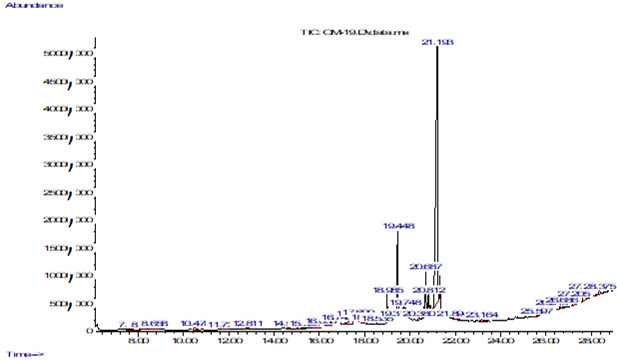
GC-MS information of *P. austroarabica* extract

Table 2, Figure 3 & Figure 4 shows the leaves aqueous extract of *P. austroarabica* inhibited the growth of *Staph. Aureus* by 6.3±0.12 mm and 24±0.43mm, 15±0.56 respectively for seed, leaf and stem at concentrations 50 µl. However, the same concentrations inhibited the growth of *E. coli* by 25±0.75, 0.00 mm and 24±0.18 mm, following the same order. Different superscript letters indicate means that are significantly different at level (p<0.05). Minimal inhibitory concentrations (MIC) of *P. austroarabica* ethanolic extracts against the tested microorganisms were 1.5, 1.6 and 1.5 respectively for seed, leaf and stem against *Staph. Aureus* and were 1.2, 0.00 and 1.2 respectively for seed, leaf and stem against *E. coli* shown Table 2.

## Gas chromatography-mass spectrometry (GC-MS):

The chromatograms, of *P. austroarabica* ethanolic extracts obtained using gas chromatography-mass spectrometry (GC-MS), are depicted in Figure 1. The data exhibit discernible peaks, indicating the presence of 20 identifiable chemical compounds. Table 1 displays the compounds and their respective gas chromatography-mass spectrometry (GC-MS) information. From the table, it can be seen that the percentage order of chemical compounds was as follows: Estragole (29.69%) > Benzene, 1-methoxy-4-(1-propenyl)- (11.83%) > eicosane (10.56%) > 2-nonacosanone (8.85%) > benzenepropanoic Benzeneacetic acid, 4-(1,1-dimethylethyl)-, methyl ester, DL-Arabinitol (7.26%) > Decanoic acid, 3-methyl-, 9,17-Octadecadienal, (Z)- (4.61%) > 9-Octadecenoic acid, (E)- (4.34%) > Hexadecanoic acid, methyl ester, Oxiraneundecanoic acid, 3-pentyl-, methyl ester, trans- (3.42%) > n-Hexadecanoic acid (2.99%) > 10-Nonadecanone, 9-Octadecenoic acid (Z)-, methyl ester, 5-Eicosene, (E)- (2.53%) > cis-Vaccenic acid (2.48%) > 9,12-Octadecadienoic acid (Z,Z)- (1.68%) > Octadec-9-enoic acid (1.67%) > 6-Octadecenoic acid, (Z)- (1.46%) > 6-Octadecenoic acid 1.03%) > n-Propyl 11-octadecenoate (1.01%) > 2-Dodecen-1-yl(-)succinic anhydride (0.93%).

## Discussion:

The present study revealed that *P. austroarabica* processed cytotoxic and anti-cancer properties. These findings were agreed with previous studies. Worldwide, including Saudi Arabia, mistletoe is regarded as a 'cure all' medicinal plant [20]. *Oncocalyx schimperi* has been identified as a species of medicinal plant [21]. In Yemen, *P. curviflorus* stems are used to treat cancer [22]. Additionally, *P. curviflorus* is employed in Saudi Arabian folk medicine to treat diabetes [23, 24]. According to a recent study, *P. curviflorus*, which was collected in Saudi Arabia, may hold promise for the creation of prostate cancer chemotherapeutics [25, 26]. In addition, [27] performed a study to identify and catalog the medicinal plants used in the traditional system in various regions of Saudi Arabia to treat various livestock ailments. The study concluded that *O. schimperi*, *P. curviflorus* and *T. globiferus* are used to treat flatulence disease, to increase lactation and for removal of placenta in cow, camels and goats. Nevertheless, mistletoe rarely causes side effects when used in the recommended dosages. However, the side effects are more likely to occur when it is used in excessive doses. The side effects include headache, fatigue, chills, nausea, vomiting, upset stomach, fever, pruritus (itchy skin), and chills. These results in line with the reported cytotoxic activity of *P. austroarabica* extract against MCF-7 [28] and another breast cancer cells (MDA-MB-231) which may elucidate the cytotoxic properties of this species against breast cancer cells.

Moreover, earlier studies on *Plicosepalus* genus revealed several biological activities like antioxidant, antihepatotoxic, anti-diabetic, antiviral, antimicrobial and cytotoxic activities [29]. The use of *T. globiferus* as antifungal agent is potential as the methanol leaf extract and its fractions showed excellent antifungal activity against some selected fungal species including *Candida albicans*, *Trychophyton mentagrophytes*, *Trychophyton rubrum* and *Aspergillus niger* [30]. In Saudi Arabia, *O. glabratus* aerial parts were studied, and some compounds that demonstrated cytotoxicity, antiviral activity against the hepatitis B virus, and anti-diabetic activity were isolated [31]. The present investigation showed that the leaves aqueous extract of *P. austroarabica* inhibited the growth of *Staph. Aureus* and *E. coli*. A variety of bacteria were used to test the activity of *Viscum* species *in vitro* [7]. It was found that the extracts' antibacterial action was more potent toward gram-negative bacteria compared to gram-positive bacteria [32] [33]. *Viscum* spp.'s antifungal activity was frequently examined on species of Candida, which are important microbes responsible for crucial morbidity as well as mortality in seriously ill hospitalized individuals [34]. Nacsa-Farkas carried out a research experiment in which twelve species of Candida were tested, with *Candida inconspicua* being the most sensitive [35]. Mistletoe's antiviral properties have not yet been thoroughly studied. The development of the human *parainfluenza virus* type 2 (HPIV-2) *in vitro* cells were found to be prevented by the water-based extract of leaves from *Viscum album* that develops upon lime trees. It was suggested that mistletoe might be helpful as an additional therapy for individuals with human immunodeficiency virus (HIV) because of its strong immune-boosting effects. In Saudi Arabia, the six different mistletoe species from four different genera of the family Loranthaceae that naturally grow in various locations were investigated for their antimicrobial activity by Waly [8]. The antimicrobial effectiveness of the extract of mistletoe plant was evaluated against gram-positive (*Staphylococcus aureus* and *Bacillus subtilis*), gram-negative (*Escherichia coli*, *Pseudomonas aeruginosa*, and *Salmonella typhi*), and yeast (*Candida albicans*) bacterial and yeast strains. The antimicrobial findings indicated that, depending on the bacterial strain and the used concentration, methanolic extracts of the six Loranthaceae species showed varying degrees of growth inhibition. It was concluded that these plants of *Plicosepalus*, *Phragmanthera*, *Tapinanthus* and *Oncocalyx* should be taken in consideration, as reported in folk medicine, as potential. Regarding the mistletoe pharmacological value, over decades, various preparations of mistletoe extract developed, including aqueous, hydroalcoholic, and ethanolic extracts [38][36][37]. When using whole extracts rather than just purified lectins and toxins, the observed pharmaceutical effects are typically easier to identify [38]. Its therapeutic effects have been shown to be cytotoxic, an apoptosis inducer, and an immunomodulator. In a rodent model, Parvez and Rishi confirmed the hypoglycemic salutation of *O. glabratus* and showed that its newly isolated flavan derivative Oncoglabrinol C activated PPARα/γ in liver cells (HepG2) [39]. The ability of Oncoglabrinol C to reverse endothelial oxidative and apoptotic damage as well as to activate hepatic CYP3A4 was demonstrated. This justifies additional research into Oncoglabrinol C and related compounds in order to create efficient and secure medications for cardiovascular disorders linked to diabetes [40]. The shoots of *P. scurviflorus* growing in Saudi Arabia were used to isolate naphthalene, the flavanol curviflorin, catechin, and quercetin. There have been reports of naphthalenes in liverworts, plants, insects, and fungi. According to Ibrahim and Mohamed (2017), naphthalenes have a variety of bioactivities, including antimicrobial, antioxidant, cytotoxic, anti-inflammatory, anti-platelet aggregation, and antiprotozoal. There are several medications that contain naphthalene, including nafacillin, naftifine, tolnaftate, and terbinafine, which are essential for managing microbial infection [41]. The *T. globiferus* plant showed anti-inflammatory, immunomodulatory, antioxidant, osteoprotective, and hepato-nephroprotective properties in addition to antitrypanosomal activity and anticonvulsant activity, according to some pharmacological studies on the plant growing on various host species [42]. The use of homeopathic medications made from plant extracts, which have been experimentally studied in Saudi Arabia and other nations, is highlighted in this context. The literature does not contain any information about the effects of commercial formulations of prepared mistletoe, which contain very small amounts of their active ingredients. With respect to the results of mistletoe phytochemical constituents, it is difficult to pinpoint every active phytochemical compound found in mistletoe plants. Proteins, polysaccharides, oligosaccharides, steroids, triterpenes, flavonoids, alkaloids, and lipophilic molecules are just a few of the many different substances that have been described [43, 44]. From various *Plicosepalus* species, flavonoids, phenolic acids, triterpenes, sterols, and sesquiterpene lactones have been isolated. *Plicosepalu curviflorus* has been used to isolate flavane gallates, triterpenes, and sterols [45, 46]. Alkaloids, flavonoids, polyphenols, and tannins are among the bioactive metabolites found in the aqueous extracts of the entire *T. globiferus* plant [47]. Three flavan derivatives, oncoglabrinol A, oncoglabrinol B, and oncoglabrinol C, were isolated from the active ethyl acetate extract of *O. glabratus* after chemical analysis of aerial parts collected from Saudi Arabia looked into the phytochemical components of the mistletoes of Saudi Arabia. The methanolic extracts' phytochemical screening revealed that flavonoids, steroids, and/or terpenoids were among their main constituents. Alkaloids, cardenolides, and saponins, on the other hand, were not found in any of the extracts that were examined. Anthraquinones and tannins are only weakly accumulated by *P. austroarabica*. The shoots of *P. scurviflorus* growing in Saudi Arabia were used to isolate the flavonoids catechin and quercetin, the naphthalene glycoside curviflorside, and the flavanol curviflorin [29]. According to Abdallah *et al.* [48], O-caffeoyl quinic acid conjugates and oléanane triterpenes were the main compounds that might be responsible for antihyperglycemic effect of *V. schimperi*.

## Conclusion:

*P. austroarabica* showed a remarkable activity *via* suppressing the cell's survival. In terms of IC50 (concentration equivalent to a survival rate of 50%), MCF-7 breast cancer cells were more sensitive to *P. austroarabica* extract.) *P. austroarabica* has DPPH antioxidant properties. The antioxidant activity was increased along with increment of extract concentrations. *P. austroarabica* has antimicrobial activities against of *S. aureus* and *E. coli*. Different superscript letters indicate means that are significantly different at level (p<0.05). Minimal inhibitory concentrations (MIC) of *P. austroarabica* ethanolic extracts against the tested microorganisms were 1.5, 1.6 and 1.5 respectively for seed, leaf and stem against *Staph. Aureus* and were 1.2, 0.00 and 1.2 respectively for seed, leaf and stem against *E. coli*.

## Data availability:

Data are available within the manuscript

## Author contributions:

All of the authors (Moodi S. A. Alsubeie, Ahmed A. Alghamdi, Nasir A. Ibrahim, Nosiba S. Hamed, BS Al-ammari, Awadallah B. Dafaallah and Vajid Nettoor Veettil) contributed equally to data collection, drafting, processing, writing, editing and reviewing article.

## Funding:

This work was supported and funded by the Deanship of Scientific Research at Imam Mohammad Ibn Saud Islamic University (IMSIU) (grant number IMSIU-RG23139).

## Figures and Tables

**Table 1:** The IC50 values of *P. austroarabica* in various cancer cells

**Sample**	**Cell lines and IC50(µg/ml)**	
	**A549**	**MCF-7**
*P.austroarabica*	88.76 ± 0.75	76.15 ± 1.06
Doxorubicin	1.3 ± 0.05	0.95 ± 0.04

**Table 2:** Antibacterial activity (mm) of *P. austroarabica* ethanolic extracts.

**Organism**	**Inhibition Zone of seed**	**Inhibition Zone of leaf**	**Inhibition Zone of stem**
*S. aureus*	14±0.24	24±0.43	15±0.56
*E. coli*	25±0.75	0	24±0.18

**Table 3:** Minimal inhibitory concentrations (MIC) of *P. austroarabica* ethanolic extracts against the tested microorganisms

**Tested organism**	**Seed extract**	**leaf extract**	**stem extract**
*S. aureus*	1.5	1.6	1.5
*E. coli*	1.2	0	1.2

**Table 4:** GC-MS information of *P. austroarabica* extract

**No**	**Compound**	**RT (min)**	**Peak area (%)**	**Mol Weight (amu)**	**Molecular formula**
1	Estragole	10.478	0.35	148.089	C_10_H_12_O
2	Benzene, 1-methoxy-4-(1-propenyl)-	11.735	0.325	148.089	C_10_H_12_O
3	Benzeneacetic acid, 4-(1,1-dimethylethyl)-, methyl ester	14.575	0.219	206.131	C_13_H_18_O_2_
4	DL-Arabinitol	16.746	0.782	152.068	C_5_H_12_O_5_
5	Decanoic acid, 3-methyl-	17.44	0.394	186.162	C_11_H_22_O_2_
6	9,17-Octadecadienal, (Z)-	18.091	0.175	264.245	C_18_H_32_O
7	9-Octadecenoic acid, (E)-	18.535	0.169	282.256	C_18_H_34_O_2_
8	Hexadecanoic acid, methyl ester	18.985	0.113	270.256	C_17_H_34_O_2_
9	Oxiraneundecanoic acid, 3-pentyl-, methyl ester, trans-	19.317	0.307	312.266	C_19_H_36_O_3_
10	n-Hexadecanoic acid	19.448	0.175	256.24	C_16_H_32_O_2_
11	10-Nonadecanone	19.748	0.475	282.292	C_19_H_38_O
12	9-Octadecenoic acid (Z)-, methyl ester	20.687	0.163	296.272	C_20_H_38_O_2_
13	5-Eicosene, (E)-	20.812	0.169	280.313	C_20_H_40_
14	cis-Vaccenic acid	21.193	0.35	282.256	C_18_H_34_O_2_
15	9,12-Octadecadienoic acid (Z,Z)-	21.894	0.388	280.24	C_18_H_32_O_2_
16	Octadec-9-enoic acid	23.164	0.394	282.256	C_18_H_34_O_2_
17	6-Octadecenoic acid, (Z)-	25.597	0.263	282.256	C_18_H_34_O_2_
18	6-Octadecenoic acid	26.248	0.594	282.256	C_18_H_34_O_2_
19	n-Propyl 11-octadecenoate	26.686	0.319	324.303	C_21_H_40_O_2_
20	2-Dodecen-1-yl(-)succinic anhydride	27.205	0.294	266.188	C_16_H_26_O_3_
